# Children’s and adolescents’ perspectives on routine inquiry about violence in specialised outpatient care

**DOI:** 10.1186/s13104-025-07175-6

**Published:** 2025-03-21

**Authors:** Mari Brännvall, Karin Örmon, Solveig Lövestad

**Affiliations:** 1 The Västra Götaland Region Competence Center on Intimate Partner Violence, Gothenburg, Sweden; 2 Department of Health, Blekinge Institute of Technology, Karlskrona, Sweden; 3 Department of Public Health and Community Medicine, Institution of Medicine, University of Gothenburg, Gothenburg, Sweden

**Keywords:** Violence screening, Children and adolescents, Outpatient care, Mixed methods, Domestic violence

## Abstract

**Objective:**

This study explores children’s and adolescents’ experiences and opinions of routine inquiries about violence within specialised outpatient care. Utilising a mixed method with a convergent parallel design, the research combines quantitative data from 184 respondents aged 6–17 collected through survey data and qualitative interviews with four participants aged 7–14. The data presented is a byproduct of an ongoing research project that evaluates a questionnaire designed to ask children about violence.

**Results:**

Findings indicate that most children and adolescents view routine questioning about violence positively or neutrally. The study highlights the importance of healthcare professionals’ responses to disclosures of violence, emphasising that supportive and empathetic reactions can impact children’s willingness to disclose such experiences in the future. The results underscore the necessity for routine inquiries about violence in healthcare settings to ensure that affected children receive appropriate support and intervention.

## Introduction

The World Health Organization reports that half of the world’s children experience violence annually [1]. Childhood exposure to domestic violence has immediate and long-term health impacts, strongly correlating with later physical and mental health issues [2, 3], with a resulting need for specialized care. A Swedish study found that 20–30% of child and adolescent psychiatric patients had experienced family violence [4]. In addition, previous research indicates that children exposed to violence are more likely to become perpetrators or victims of intimate partner violence (IPV) and future perpetrators of child abuse, emphasizing the importance of early identification of children exposed to or engaging in violence [5, 6]. Healthcare providers are likely to encounter abused children, thus, they play a crucial role in identifying child patients exposed to violence [7]. Furthermore, in Sweden, healthcare providers are required to ask children about violence if they suspect that a child has been exposed to or has witnessed violence [8]. Research shows children are more likely to disclose violence if they have opportunities to do so, see a purpose with the disclosure, and perceive support [9]. Not asking about violence reduces disclosure [10]. According to a Swedish study, non-disclosure was often due to believing no one could help. Reporting violence seldom led to significant changes, and interventions were seen as ineffective [11].

Low disclosure rates among children in healthcare settings have an impact on the necessary support or treatment for violence [11]. A literature review highlights the feasibility of routine inquiries for domestic violence in paediatric and adolescent mental health services, emphasising the need for appropriate tools and an empathetic approach [12]. However, few self-report clinical tools for children exist, further research being therefore needed in this area [13, 14]. According to a Norwegian study, routine inquiries for domestic violence appears to be well tolerated by children and adolescents, with the majority reporting minimal or no distress [15] However, more knowledge is needed about children’s attitudes towards routine inquiry within healthcare settings, which this study aims to contribute to.

In western Sweden, a questionnaire to identify children’s (ages 4–17) experiences of violence was piloted at seven clinics from 2018 to 2021. The Questions on Violence tool for children (FOV-children) includes six questions on witnessing and experiencing physical, emotional, sexual, and digital violence, plus one for adolescents about being abusive. The FOV-Children tool is currently undergoing a validation process using the DELPHI method. This involves multiple rounds of expert consultation, where a panel of specialists is asked to provide feedback on the tool by responding to a series of questions. This article represents an early stage of that process, aiming to describe children’s opinions and experiences of routine violence assessment in specialised outpatient care.

## Main text

### Methods

A mixed method with a convergent parallel design was used. The method is beneficial when qualitative and quantitative methods are applied simultaneously. The quantitative and qualitative data are complementary and gathered about a central phenomenon comprising data collection, analyses, and, finally, the result of both quantitative and qualitative data within a specific study, in order to address distinct but related research questions [16].

### Quantitative design and sample

The instructions specified asking questions about violence to all children. Thus, the inclusion criteria were children aged 4 to 17 years who made their first visit to the clinic during the period, regardless of suspicion of exposure to violence. However, the study included only children aged 6 years and older, excluding those aged 4 and 5 years. A cross-sectional study using the FOV tool was conducted over three months in 2020. A total of 107 children, aged 6–13, and 78 adolescents, aged 13–17, answered questions on violence. One adolescent was excluded due to missing information about gender, leaving a total of 77 adolescent respondents.

Healthcare staff asked children (4–12 years) orally and adolescents (13–17 years) either orally or in writing in the examination room. All answers were recorded in the questionnaire. Out of 370 patients surveyed, 185 completed questionnaires were received, resulting in a response rate of 50.0%.

### Measurement tool and variables

For both children and adolescents, age at the interview was calculated based on birth year and interview date and categorised into three groups: 6–7, 8–10, and 11–13 years for children and 12–13, 14–15, and 16–17 years for adolescents. For 15 adolescents, missing birth year and/or interview date prevented age calculation.

**Fig. 1:**
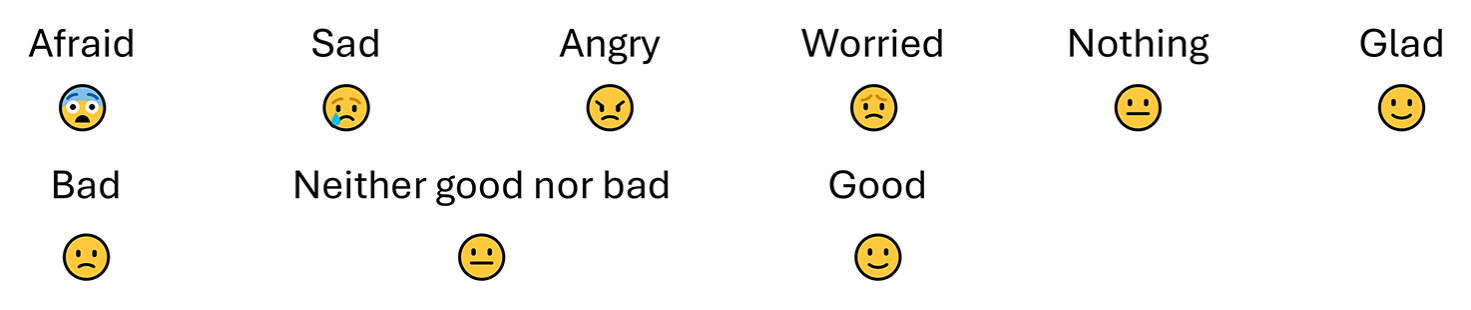
Symbols corresponding to response options in the survey for children aged 4–12 Years

Children aged 6 to 13 were asked about their feelings on being questioned about violence, with response options visualised by corresponding facial expressions. This was to ensure that children who were not literate could still respond to the questions. Previous research suggests that visual aids can enhance visual attention, improve understanding, and facilitate the recall of information [17]. The facial expressions corresponding to the emotions were tested before measurement by a test group consisting of five children aged 5–9 years. The question ‘How did you feel when you answered the questions?’ had six response options: (1) afraid, (2) sad, (3) angry, (4) worried, (5) nothing special, and (6) glad (Fig. 1). Due to low cell frequencies, the response options afraid, sad, angry, and worried were merged into one category. The questions ‘How did you feel when you were asked if someone had hurt you or someone else?’ and ‘What do you think about adults working here asking all children if someone has hurt them or someone else?’ had three response options each: ‘bad’, ‘neither good nor bad’, and ‘good’.

Respondents aged 13 to 17 were asked about their feelings on being questioned about being abused or their own perpetration of violence. Response options were: ‘bad’, ‘neither good nor bad’, and ‘good’. They were also asked if they could answer the questions honestly. Response options were ‘yes’ and ‘no’.

### Quantitative data analysis

Analyses were computed using SPSS, version 25. Frequency (n) and prevalence (%) were calculated for feelings and opinions on being asked about violence and stratified by gender. Differences between girls and boys were calculated using the chi-squared test with the Fisher’s Exact probability test (*p* <.05) for categorical variables with expected frequency less than five. Effect size was calculated using Cramers V (V). For questions addressed to adolescents on feelings and thoughts about being questioned on violence, the chi-squared test was not applied due to violation of the assumption that at least 80% of cells have expected frequencies of 5 or more.

### Qualitative design and sample

Inclusion criteria for the qualitative interview study were children aged 4 + at a psychiatric clinic, children aged 6 + at a specialist primary healthcare centre, and children aged 6 + diagnosed with headache, stomach ache, encopresis, incontinence, or obesity at a medical centre, and participants needed to speak and understand Swedish.

### Qualitative data collection

Healthcare staff asked children, adolescents, and their accompanying parents about participation in the study. Oral and written information, appropriate for the child’s age, was provided during their first clinic visit. Consent to be contacted by researchers was either left at the reception or mailed in a prepaid envelope. The initial goal was to include 20 children in the study. Ultimately, two boys (aged 7 and 9) and two girls (aged 13 and 14) expressed interest in participating and were subsequently included in the interview study.

Three interviews were conducted in the participants’ home, and one at the clinic. The interview guide focused on experiences of being asked about violence and staff responses to disclosures. Interviews began with, ‘When you visited (name of healthcare setting), you answered questions about experiencing violence. Now I would like to talk to you about what it was like’ Conducted in autumn 2020 and spring 2021, the interviews lasted 15–23 min and were audio-recorded and transcribed verbatim.

### Qualitative data analysis

Thematic analysis, as described by Braun and Clarke [18], was utilised for this study due to its flexibility and wide applicability. The interviews were thoroughly reviewed, with relevant quotes highlighted. Initial codes were identified, refined, and organised, resulting in four themes.

## Results

### Quantitative results

#### Child respondents

In total, 107 children, 61 girls (57.0%) and 46 boys (43.0%) aged 6–13, participated in the study (Table 1). Most of the children were aged 8–10 at the interview (53.4% vs. 61.0%) (*p* =.016, V = 0.29) (Not in table).

When asked about being questioned on violence by healthcare personnel, the majority of both girls (95.1%) and boys (73.8%) answered that it was ‘good’. Although not statistically significant, 76.7% of the girls and 61.4% of the boys responded that they felt ‘nothing special’ while answering, followed by ‘glad’ (11.7% vs. 20.5%) (*p* =.24).

**Table 1:** Feelings and opinions among children aged 6–13 years on answering questions about violence. Presented as frequencies (n) and valid proportions (%). Total *N* = 107

Questions	Girls *N* = 61		Boys *N* = 46		Chi^2^ tests
**How did you feel while answering the questions?**	***n***	**Valid** ^**a**^ **%**	***n***	**Valid** ^**a**^ **%**	
Afraid, sad, worried, angry	7	11.7	8	18.2	0.24
Nothing special	46	76.7	27	61.4	
Glad	7	11.7	9	20.5	
**How did you feel when you were asked if someone had hurt you or someone else?**					
Bad	2	3.3	5	11.6	0.24
Neither good nor bad	27	45.0	16	37.2	
Good	31	51.7	22	51.2	
*Missing*	*1*		*3*		
**What do you think about adults working here asking all children if someone has hurt them or someone else?**					
Bad	0	0.0	3	7.1	***NA***
Neither good nor bad	3	4.9	8	19.0	
Good	58	95.1	31	73.8	
*Missing*	*0*		*4*		

^a^ Valid proportion, missing values excluded

^NA^ Not applicable due to violation of the assumption of at least 80% of cells with expected frequencies of 5 or more

### Adolescent respondents

A total of 77 adolescents (51 girls, 26 boys) aged 12–17 responded to the questionnaire (Table 2). Most girls (41.2%) and boys (38.5%) were 14–15 years old (*p* =.048, V = 0.31) (Not in table).

When asked about being questioned on perpetrating violence, 52.9% of the girls and 53.8% of the boys felt ‘good’. Furthermore, 90.2% of the girls and 84.6% of the boys responded that they felt ‘good’ about staff asking all children and adolescents about violence. Almost all girls (90.2%) and all boys (100.0%) responded that they felt they could answer honestly.

**Table 2:** Feelings and opinions among youth aged 12–18 years on answering questions about violence. Presented as frequencies (n) and proportions (%). Total *N* = 77

Questions about being asked about violence	Girls *N* = 51		Boys *N* = 26	
	*n*	%	*N*	%
**How did you feel about being asked if you had experienced violence?**				
Bad	0	0.0	0	0.0
Neither good nor bad	17	33.3	10	38.5
Good	30	58.8	16	61.5
*Missing*	*4*	*7.8*	*0*	*0.0*
**How did you feel about being asked if you had subjected someone else to violence?**				
Bad	1	2.0	0	0.0
Neither good nor bad	18	35.3	12	46.2
Good	27	52.9	14	53.8
*Missing*	*5*	*9.8*	*0*	*0.0*
**How do you feel about staff asking all children and young people who visit this centre about violence?**				
Bad	0	0.0	0	0.0
Neither good nor bad	0	0.0	4	15.4
Good	46	90.2	22	84.6
*Missing*	*5*	*9.8*	*0*	*0.0*
**Could you answer the questions about violence honestly?**				
No	0	0.0	0	0.0
Yes	46	90.2	26	100.0
*Missing*	*5*	*9.8*	*0*	*0.0*

### Qualitative results

#### Emotions related to being asked about abuse

The informants generally reported feeling comfortable when asked about their experiences of abuse, finding the questions relatively easy to answer. The analysis shows that the questions were expected, reflecting the caregiver’s aim to assess well-being. However, one informant first found it a bit strange to be asked about abuse when seeking care for anxiety. The written inquiry was described as creating a sense of alienation:

Well, getting a piece of paper will hardly be helpful, I think. I think in that case you should do it, well, orally or how to say. Because it’s as if it becomes, well, it becomes terribly, not genuine. You sort of, here’s a piece of paper, write on it, like.

The informant thus perceived a lack of genuine concern for her abuse experiences due to the casual distribution of the questionnaire. She would have preferred oral communication for its more personal nature.

### Emotional and relational barriers to disclosure

The adolescents found it particularly difficult to discuss sexual violence, or their own abusive behaviour, due to a reluctance to admit wrongdoing. Disclosing abuse by a family member was especially challenging. One reason cited was the potential consequences for oneself and others, which reflects the complexity of navigating feelings of loyalty in familial relationships:

That would’ve been much harder because it’s after all someone you’re very close to, even though you’re not that close it’s after all your family. And then you don’t want to cause problems for that person, perhaps.

Reporting domestic abuse was challenging due not only to emotional ties and a desire to avoid harming the abusive family member, but also to a need to protect one’s privacy.

Well, I felt more that maybe everyone doesn’t need to know everything. That you may want to keep some things to yourself that everyone doesn’t know about.

### Supporting children to disclose

The informants endorsed the practice of inquiring about abuse among all patients visiting the clinics. This approach enables staff to identify child abuse, which might be difficult for children to disclose.

But it’s good that it’s raised, if people have been [abused] and haven’t dared to say it and maybe think that now someone asks about it.

One informant highlighted that children do not disclose abuse unless they are specifically asked about it.

### The importance of being validated

All informants reported having experienced or witnessed psychological, physical, or sexual abuse either at school or online, but these experiences were not perceived as having significantly impacted their lives. However, they noted that after affirming the answer to one question, the staff often moved promptly to the next without further discussion. While most informants did not find this approach problematic, expressing relief that someone was now aware of their experiences, one participant described how the lack of acknowledgement or response from the staff led to feelings of having shared personal information without receiving any meaningful recognition or support in return:

You know, then it feels a little as if she’ll maybe give it to someone else and as if I may not get anything out of it, sort of. That it’s just people who’ll learn about it and think ‘okay’, like. […] I mean, that they maybe think that ‘now she’s written it. Now I don’t need to talk to her about it, because now she’s written it here and now, we have the answers’, or something like that.

One interview revealed a wish that staff had probed further and explored the need for additional support. While the abuse was not perceived as a significant issue by that informant, greater interest and engagement from the staff was desired. This highlights the critical role of the staff’s response when children and adolescents disclose experiences of abuse, as it can significantly influence their sense of being heard and supported.

## Discussion

The study’s findings suggest that children and adolescents do not object to answering questions about violence when seeking specialised outpatient care. Nearly all respondents felt that being asked about violence was either ‘good’ or ‘neither good nor bad’. Consistent with other research [15], few children perceived the questioning negatively. However, it is possible that some children who answered the questions may not have fully associated the symbols with the intended emotions. Children’s cultural background may have also influenced how they interpreted facial expressions, which in turn may have affected how they responded. Furthermore, health care personnels attitudes and fears of asking about violence [19] may have influenced on how children responded about their feelings on being questioned about violence. Most respondents appreciated the routine practice of inquiring about violence at the clinics. As indicated in other research [10], the responses of adults to children’s disclosures of violence significantly impact the children’s experiences of sharing. It is crucial that children’s experiences of violence are acknowledged and not met with silence. Furthermore, adults’ responses have been shown to influence children’s willingness to disclose violence in the future [10].The low response rate and the small sample size may have affected the external validity, limiting the generalisability to a broader population of children, including those from minority groups, undocumented children, and children severely exposed to violence at home. The results must therefore be interpreted with caution. The studies contribution to clinical practice highlights the importance of routine inquiry about exposure to violence when children and adolescents seek medical care. Furthermore, healthcare providers should acknowledge and validate children’s disclosures, as sharing such experiences may evoke feelings of disloyalty. A more extensive future study could provide deeper insights into this phenomenon and its implications for clinical practice.

### Limitations

- Four children and adolescents were interviewed, which is too few to identify patterns.
- The qualitative sample was small, which influences the depth of thematic analysis.
- Covid-19 pandemic may have affected recruitment, as there was widespread fear of infection and periodic restrictions on having face-to-face meetings.
- The response rate for the survey was 50% which influences on the generalisability of the results.
- The self-reported data in this study may have been influenced by social desirability bias, leading to the underreporting of exposure to violence perpetrated by caregivers.
- Answers may be less negative due to staff being present when questionnaires were answered.

## Data Availability

No datasets were generated or analysed during the current study.
